# Intraoperative specimen ultrasonography as a tool for real-time margin assessment in breast-conserving surgery

**DOI:** 10.3389/fonc.2026.1947438

**Published:** 2026-09-10

**Authors:** Burak Celik, Safa Toprak, Mustafa Irfan Sokmen, Ece Erenler, Orhan Agcaoglu, Ece Dilege

**Affiliations:** 1 Koç University School of Medicine, Department of General Surgery, Istanbul, Türkiye; 2 Koç University School of Medicine, Istanbul, Türkiye

**Keywords:** breast cancer, breast conserving surgery, intraoperative specimen ultrasonography, margin assessment, re-excision

## Abstract

**Background:**

Adequate margin clearance is a key determinant of local control after breast-conserving surgery (BCS), yet dedicated intraoperative margin assessment is not available in all settings. Intraoperative specimen ultrasonography (IOUS) is widely accessible and can be performed by the surgeon. We evaluated the diagnostic accuracy of IOUS for detecting close or positive margins against permanent pathology, and its concordance with intraoperative pathological assessment.

**Methods:**

In this prospective observational study, 60 consecutive patients undergoing BCS for invasive breast cancer were enrolled. The surgeons measured tumor-to-margin distances in six orientations by IOUS, while the pathologist, blinded to the measurements, performed gross assessment and the intraoperative re-excision decision. The permanent paraffin margin of the primary specimen served as the reference standard, with a close margin defined *a priori* as <2 mm. Diagnostic performance was assessed at the patient level using ROC analysis and at the 2 mm threshold, and at the margin level using generalized estimating equations to account for within-patient correlation. Agreement was evaluated with Bland–Altman analysis and the intraclass correlation coefficient (ICC).

**Results:**

Among 60 patients a positive margin was present in 5 (8.3%) and a close margin (<2 mm) in 17 (360 margins) (28.3%). The minimum IOUS margin discriminated close margins with an AUC of 0.779 (95% CI 0.637–0.921). At the patient level, using the 2 mm threshold, IOUS correctly identified 10 of 17 close-margin patients, with a sensitivity of 58.8% and specificity of 86.0%. Compared with the pathologist’s re-excision recommendation (sensitivity 76.5%, specificity 76.7%), no significant difference was detected (McNemar, *p = 0.167*), although the study was not powered for equivalence; IOUS was less sensitive but more specific. At the margin level, each 1 mm increase in the IOUS margin distance was associated with a 36% reduction in the odds of that margin being in the close (OR 0.64, 95% CI 0.50–0.82; *p <0.001).*

**Conclusion:**

Surgeon-performed IOUS provides intraoperative margin assessment with no significant difference from the pathologist’s evaluation. It may serve as a practical, accessible adjunct where immediate intraoperative pathology is not available, though it cannot fully replace definitive histopathological assessment.

## Introduction

Breast cancer (BC) remains the most common cancer in women, and the adoption of effective early detection strategies continues to provide excellent survival outcomes (1). Early-stage diagnosis allows tumors to be detected at smaller sizes, thereby making breast-conserving surgery (BCS) an appropriate and often preferred surgical option for many women. BCS was associated with better long-term physical well-being, highlighting its potential quality-of-life advantage in BC as well (2).

Beginning in the early 1970s, as the oncologic safety of BCS was systematically investigated, the incorporation of radiotherapy (RT) emerged as a major component of treatment, establishing BCS as a safe and effective surgical approach with durable local control. In the NSABP B-06 trial, patients who underwent BCS without RT experienced a local recurrence rate of 39.2%, which decreased markedly to 14.3% with the addition of RT (3). In the modern era, BCS was associated with low local recurrence rates, with a 5-year incidence of approximately 4–5% among patients treated with contemporary multimodal therapy. Higher local recurrence rates were primarily observed in patients younger than 50 years, identified as an independent risk factor, and in those with triple-negative tumor biology. Hormone receptor–positive subtypes demonstrated more favorable local control. However, tumor size was not independently associated with local recurrence, in contrast to findings reported in earlier studies (4). On the other hand, margin status has consistently been identified as the strongest predictor of local recurrence (3). Based on the available evidence, the Society of Surgical Oncology (SSO) and the American Society for Radiation Oncology (ASTRO) published a consensus guideline in 2014 addressing optimal surgical margin management (5). This guideline concluded that positive surgical margins, defined as “ink on invasive carcinoma”, were associated with an approximately twofold increase in the risk of local recurrence compared with negative margins (6). Current international guidelines are consistent in defining negative surgical margins in invasive BC: the National Comprehensive Cancer Network (NCCN) defines a positive margin as “ink on tumor”, while European Society for Medical Oncology (ESMO) recommends that no tumor be present at the inked margin, a principle that is also endorsed in the latest guidelines by American Society of Clinical Oncology (ASCO) and American Society for Radiation Oncology (ASTRO) (7–9).

Several methods are available for assessing surgical margins, including macroscopic evaluation, frozen section analysis, touch prep analysis, specimen radiography, and intraoperative specimen ultrasonography (IOUS) (10). Although these conventional techniques have the potential to reduce positive surgical margins, several challenges limit their widespread use, including the time required, the technical difficulty of intraoperative pathological assessments, and the limited availability of intraoperative pathology services.

Given the widespread availability of ultrasound (US) in almost all medical centers and its ability to be performed intraoperatively by surgeons, the primary aim of this study was to evaluate the real-time intraoperative diagnostic accuracy of IOUS for detecting close or positive margins, and the secondary aim was to assess whether IOUS could support intraoperative re-excision decisions during BCS.

## Methods

### Patients

This institutional review board–approved study (Koç University Institutional Review Board (2025.010. IRB2.006) was a prospective observational cohort study conducted between August 2023 and December 2025. The sample size was calculated using G*Power (version 3.1) for a two-tailed paired-samples comparison (α = 0.05, power = 90%), based on an assumed moderate effect size (Cohen’s d = 0.5) in the absence of directly comparable prior data, yielding a minimum required sample of 55 patients (11). This calculation was based on a paired-means comparison and does not directly correspond to the primary diagnostic endpoints of this study (diagnostic accuracy and the McNemar comparison of paired binary tests). Consequently, the diagnostic accuracy estimates are precision-limited by the number of close-margin events, and the reported confidence intervals should be interpreted accordingly.

We prospectively evaluated 60 consecutive patients undergoing BCS for unifocal or multifocal BC, with surgical specimen margins assessed intraoperatively by the surgeon using US and by the pathologist during gross examination, who was blinded to the ultrasonographic measurements. All patients had a solid breast mass diagnosed as invasive carcinoma by core needle biopsy. Patients with microcalcifications within or adjacent to the tumor underwent additional margin assessment using specimen radiography. In cases of multifocal disease, US for surgical margin assessment was applied to the largest tumor. Patients who had received neoadjuvant therapy or had a previous history of BC treatment were excluded from the study.

### Surgery and margin assessment

All surgical procedures were performed under general anesthesia by three experienced breast surgeons. The surgical technique of BCS (lumpectomy or quadrantectomy) was selected based on tumor size and breast volume, and oncoplastic techniques, including reduction mammoplasty, were employed in appropriately selected patients. Wire localization was used for all non-palpable tumors.

**Figure 1:**
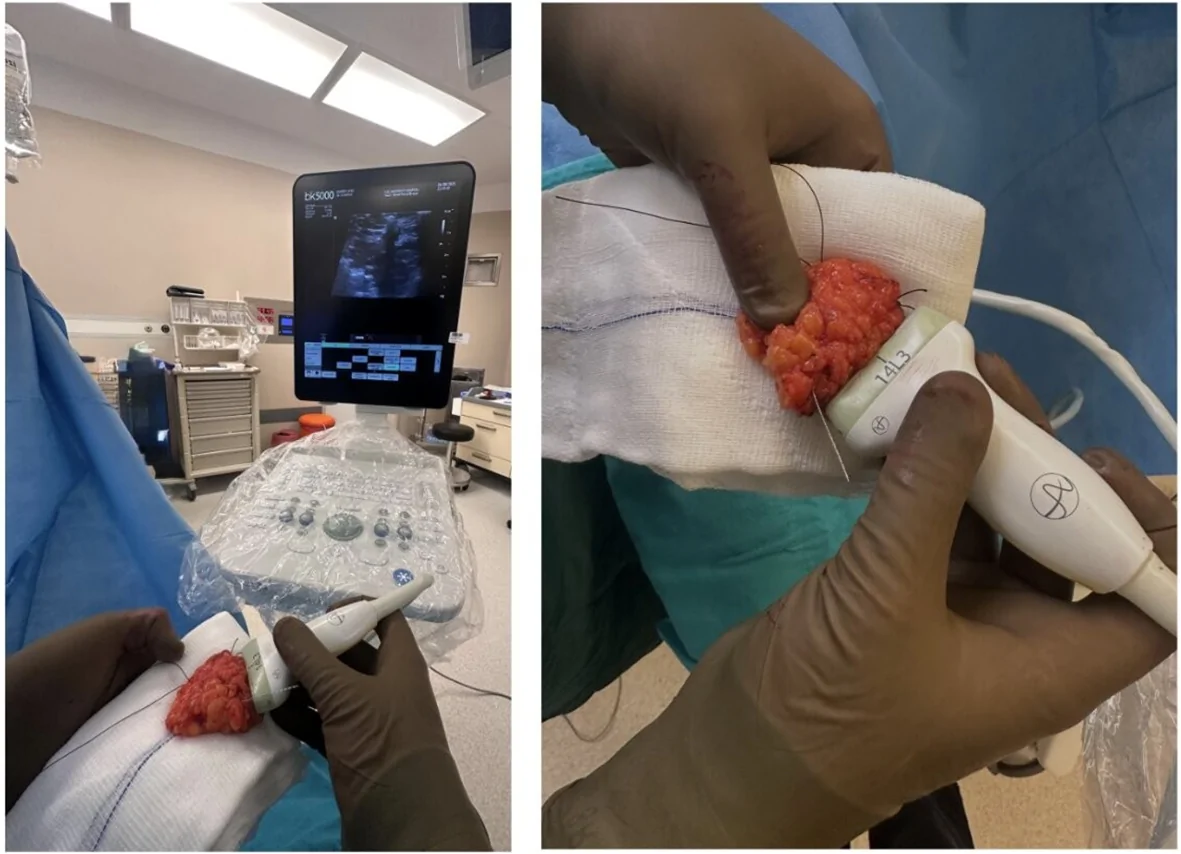
Intraoperative *ex vivo* specimen ultrasonography. The freshly excised lumpectomy specimen is scanned on the operative field immediately after removal. The surgeon applies the ultrasound probe directly to the specimen to measure the tumor-to-margin distance in each orientation, enabling real-time intraoperative margin assessment.

**Figure 2:**
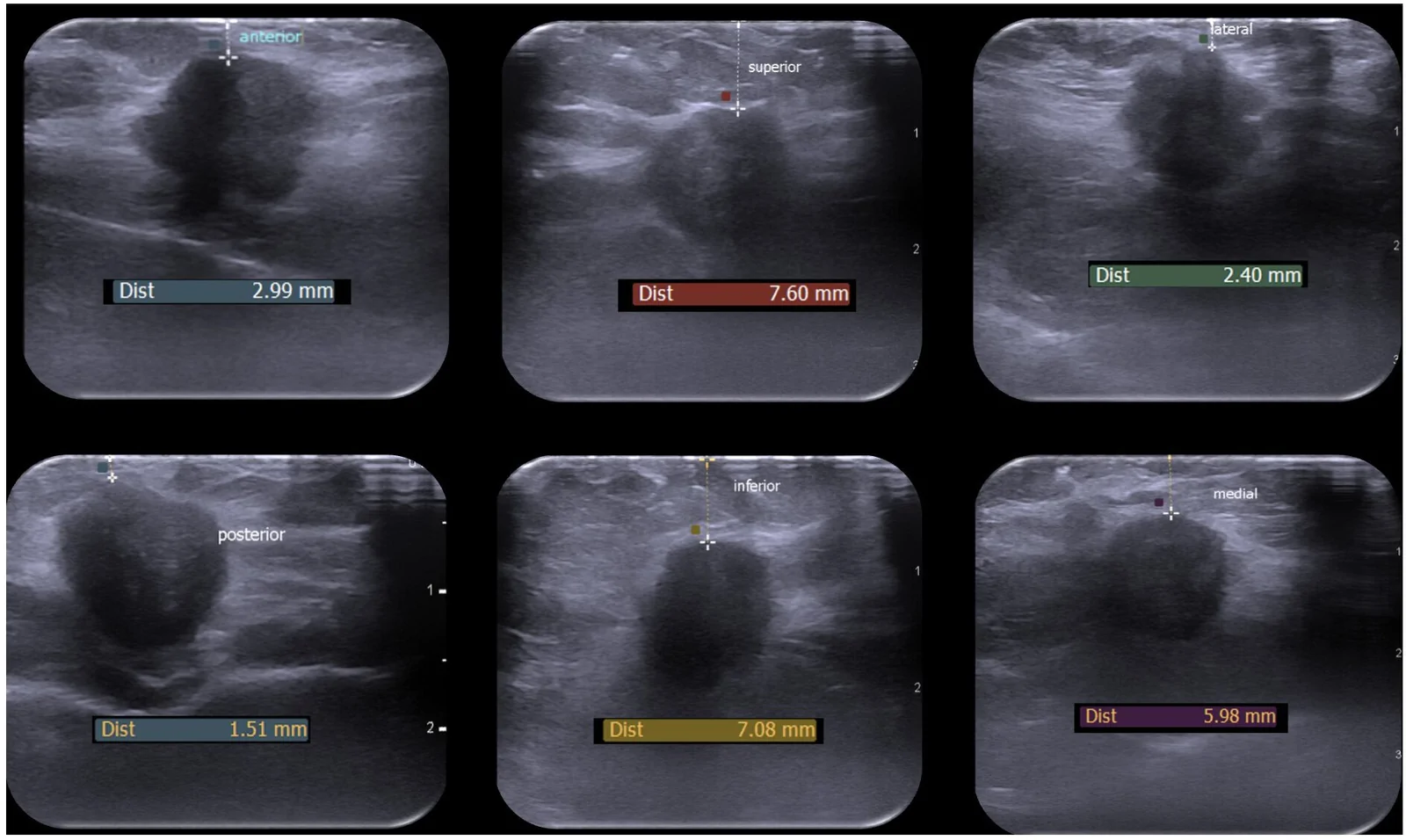
Ultrasound-based measurement of the distance between the specimen margin and the tumor at all margins (anterior, posterior, superior, inferior, lateral, and medial).

Following completion of BCS, the specimen margins were oriented with silk sutures to facilitate pathological orientation: “superior short, lateral long, and one short and posterior one long”. Ex vivo specimen US was performed by two surgeons using a “BK 5000 ultrasound system” equipped with a 14–3 MHz linear array transducer. Normal saline was used to interface with the specimen. Compression was applied to the specimen, sufficient to visualize the tumor. The degree of compression was not standardized or quantified, which represents a potential source of measurement variability and is addressed in the limitations (**Figure 1**). Subsequently, the distances from the tumor to the anterior, posterior, superior, inferior, medial, and lateral margins of the specimen were measured in millimeters (mm) and recorded individually (**Figure 2**).

After the measurements were completed, the specimen was inked by the pathologist according to a standardized color-coding protocol for orientation. Then, a macroscopic assessment of the surgical margins was performed. All margins identified as having ink on the tumor or assessed to be within 1 mm were re-excised based on the pathologic evaluation during surgery. Re-excision was not performed based on the specimen’s US findings alone. If a positive margin was identified on final pathological examination after surgery, the patient underwent reoperation for margin re-excision. The comparison of margin measurements obtained by US and pathology was performed, excluding re-excisions.

### Data analysis

The permanent paraffin margin of the primary specimen served as the reference standard. A positive margin was defined as tumor at the inked surface (“ink on tumor”; 0 mm), and a close margin was defined *a priori* as a minimum paraffin margin below 2 mm, a threshold chosen on clinical grounds consistent with established margin guidelines. Because positive margins were infrequent (n = 5), the close margin (<2 mm) was pre-specified as the primary reference for diagnostic accuracy on clinical grounds, whereas the positive-margin definition was retained as a secondary, clinically relevant endpoint. Notably, this pre-specified 2 mm threshold also coincided with the Youden-optimal IOUS cut-off identified in the ROC analysis. The same 2 mm value was used both to define the reference outcome (paraffin <2 mm) and as the IOUS test cut-off; these were selected independently, and neither was derived from the other.

Analyses were performed at two levels. At the patient level, the smallest of the six directional margins was taken as the minimum margin, providing one independent observation per patient. IOUS was assessed both as a continuous measurement, using receiver operating characteristic (ROC) analysis with the area under the curve (AUC) and 95% confidence interval (CI), and as a dichotomous test at 2 mm. The pathologist’s intraoperative re-excision recommendation was analyzed as a second binary test against the same reference. Sensitivity (the proportion of close margins correctly identified by IOUS), specificity (the proportion of clear margins correctly identified by IOUS), positive predictive value (PPV; the probability of a close margin when IOUS was positive), negative predictive value (NPV; the probability of a clear margin when IOUS was negative), and accuracy were reported with Wilson 95% CIs, and the two tests were compared using the McNemar test. As smaller IOUS margins correspond to a higher likelihood of an inadequate margin, the ROC direction was set accordingly.

At the margin level, all 360 margins (60 patients × 6 directions) were analyzed using generalized estimating equations (GEE) with a binomial distribution, logit link, and an exchangeable working correlation structure, with patient as the subject variable to account for within-patient clustering of the six margins. Two GEE models were fitted, both with a close paraffin margin (<2 mm) as the outcome: the first using the IOUS margin as a continuous predictor, and the second using the pathologist’s directional re-excision decision as the predictor. Odds ratios (OR) with 95% Wald CIs were reported.

Agreement between IOUS and paraffin margins was assessed using Bland-Altman analysis and the intraclass correlation coefficient (ICC; two-way mixed-effects model, absolute agreement, single measures). Because differences between methods varied significantly with measurement magnitude (regression of the difference on the mean, *p < 0.001*), regression-based 95% limits of agreement were used. All analyses were performed in IBM SPSS Statistics (version 28.0); a two-sided p-value below 0.05 was considered significant.

## Results

### Patient level

A total of 60 patients were included in the study, and data were obtained from evaluating 360 surgical margins, with 6 margin assessments per tumor specimen.

The mean age was 56.95 ± 13.55 years. Nineteen patients (31.7%) were premenopausal, while 41 patients (68.3%) were classified as perimenopausal or postmenopausal. The mean tumor size was 19.9 ± 9.9 mm. Tumor size distribution showed that 9 patients (15.0%) had tumors measuring 1–10 mm, 31 patients (51.7%) had tumors measuring 11–20 mm, and 20 patients (33.3%) had tumors larger than 20 mm.

Regarding histologic grade, 6 tumors (10.0%) were grade 1, 39 (65.0%) were grade 2, and 15 (25.0%) were grade 3. The most common histologic tumor type was invasive carcinoma (NOS)/invasive ductal carcinoma, observed in 42 patients (70.0%), followed by invasive lobular carcinoma in 9 patients (15.0%) and other histologic subtypes—including mixed, tubular, and micropapillary carcinoma—in 9 patients (15.0%).

Based on immunohistochemical profiling, 56 tumors (93.3%) were hormone receptor–positive and HER2-negative, 3 tumors (5.0%) showed HER2 amplification, and 1 tumor (1.7%) was classified as triple-negative. According to pathological tumor staging, 37 patients (61.7%) had T1 disease and 23 patients (38.3%) had T2 disease.

Overall, 23 patients (38.3%) underwent re-excision. Of these, re-excision was performed intraoperatively in 21 patients (35.0%), while 2 patients (3.3%) required a second surgical procedure on **Table 1**.

**Table 1:** Descriptive characteristics of the patients and tumors.

Characteristics	Patients/Tumors (n=60)
Age, mean (SD), y	56.95 (13.55)
Menopausal Status	
Premenopausal	19 (31.7%)
Perimenopausal/Postmenopausal	41 (68.3%)
Tumor Size (Mean)	19.9 (9.9)
1–10 mm	9 (15%)
11–20 mm	31 (51.7%)
>20 mm	20 (33.3%)
Histologic Grade	
1	6 (10%)
2	39 (65%)
3	15 (25%)
Histologic Tumor Type	
Invasive carcinoma (NOS)/Invasive ductal carcinoma	42 (70%)
Invasive lobular carcinoma	9 (15%)
Others (Mixed, tubular carcinoma, micropapillary carcinoma)	9 (15%)
Immunohistochemical profile	
HR (+)/Her2 (-)	56 (93.3%)
Her2 amplified	3 (5%)
Triple Negative	1 (1.7%)
T stage	
T1	37 (61.7%)
T2	23 (38.3%)
Underwent re-excision	23 (38.3%)
Intraoperatively	21 (35%)
Second surgery	2 (3.3%)

HR, Hormone Receptor; T, Tumor.

Sixty patients were analyzed. A positive margin was present in 5 patients (8.3%); this endpoint was treated as exploratory given the small number of events. **Table 2** presents the baseline clinicopathological characteristics of the cohort by final margin status. Patients with positive-plus-close margins (<2 mm) and those with clear margins (≥2 mm) were comparable in age, tumor size, histologic type, and T stage (all p > 0.05), indicating that margin status was not confounded by baseline clinicopathological characteristics.

**Table 2:** Clinicopathological characteristics by final margin status.

Variable	Close margin (n = 17)	Clear margin (n = 43)	*p*
Age, median (range), y	49 (36–75)	58 (30–85)	0.137
Tumor size, median (range), mm	18 (6–40)	18 (6–45)	0.831
Histologic type			0.637
*Ductal/NST*	11	32	
*Lobular*	3	4	
*Other*	3	7	
T stage (T1 / T2)	11/6	28/15	1.000

Close margin, paraffin <2 mm; clear margin, ≥2 mm.

Continuous variables compared with the Mann–Whitney U test and categorical variables with the chi-square or Fisher exact test. Molecular subtype was not compared because of the small number of patients in non-luminal categories. p<0.05 was considered statistically significant.

As a continuous measurement, the minimum IOUS margin discriminated well against both references, with an AUC of 0.878 (95% CI 0.678–1.000) for a positive margin and 0.779 (95% CI 0.637–0.921) for a close margin. A close margin (<2 mm) was present in 17 of the 60 patients on final pathology. Using the 2 mm threshold, IOUS identified a minimum margin below 2 mm in 16 patients: 10 of the 17 close-margin patients were correctly identified (true positives), 7 were missed (false negatives), 6 were flagged as close but were clear on pathology (false positives), and 37 were correctly classified as clear (true negatives). This corresponds to a sensitivity of 58.8%, specificity of 86.0%, positive predictive value of 62.5%, and negative predictive value of 84.1%.

The pathologist’s re-excision recommendation identified 13 of the 17 close-margin patients, with a sensitivity of 76.5% and specificity of 76.7% (**Table 3**). No significant difference was detected between the two tests (McNemar, *p = 0.167*): results were concordant in 41 patients, while IOUS alone was positive in 6 and the pathologist alone in 13. Overall, the pathologist identified more close-margin patients than IOUS (13 vs 10), corresponding to fewer patients who would potentially require re-operation (4 vs 7), whereas IOUS produced fewer false positives (6 vs 10). The study was not powered for equivalence, and the point estimates diverged clinically, with IOUS being less sensitive but more specific than the pathologist’s recommendation.

**Table 3:** Patient-level diagnostic performance for a close margin and positive margin (paraffin <2 mm), n = 60.

Measure	IOUS (min margin <2 mm)	Pathologist re-excision
Sensitivity	58.8% (36.0–78.4)	76.5% (52.7–90.4)
Specificity	86.0% (72.7–93.4)	76.7% (62.3–86.8)
PPV	62.5% (38.6–81.5)	56.5% (36.8–74.4)
NPV	84.1% (70.6–92.1)	89.2% (75.3–95.7)
Accuracy	78.3% (66.4–86.9)	76.7% (64.6–85.6)
AUC	0.779 (0.637–0.921)	Not applicable

Values are point estimates with Wilson 95% CIs. McNemar test comparing the two tests: p = 0.167. Sensitivity (the proportion of close margins correctly identified by IOUS), specificity (the proportion of clear margins correctly identified by IOUS).

### Margin level

Of the 360 margins analyzed, 6 (1.7%) were positive (ink on tumor) and 25 (6.9%) were close (<2 mm) on final paraffin pathology. In the GEE model accounting for within-patient clustering, each 1-mm increase in the IOUS margin was associated with significantly lower odds of a close margin on final pathology (OR 0.64, 95% CI 0.50–0.82; *p <0.001*). Margins selected for re-excision by the pathologist were markedly more likely to be close on final pathology (OR 72.7, 95% CI 25.0–211.3; *p <0.001*); notably, among patients with a close margin, re-excision was never performed on the wrong margin, confirming the spatial accuracy of intraoperative pathological assessment (**Table 4**).

**Table 4:** Margin-level GEE models (360 margins; outcome = close margin <2 mm).

Predictor	OR	95% CI	p
IOUS margin (per 1 mm)	0.64	0.50–0.82	<0.001
Pathologist re-excision (this margin)	72.7	25.0–211.3	<0.001

GEE: binomial distribution, logit link, exchangeable working correlation, subject = patient.

**Figure 3:**
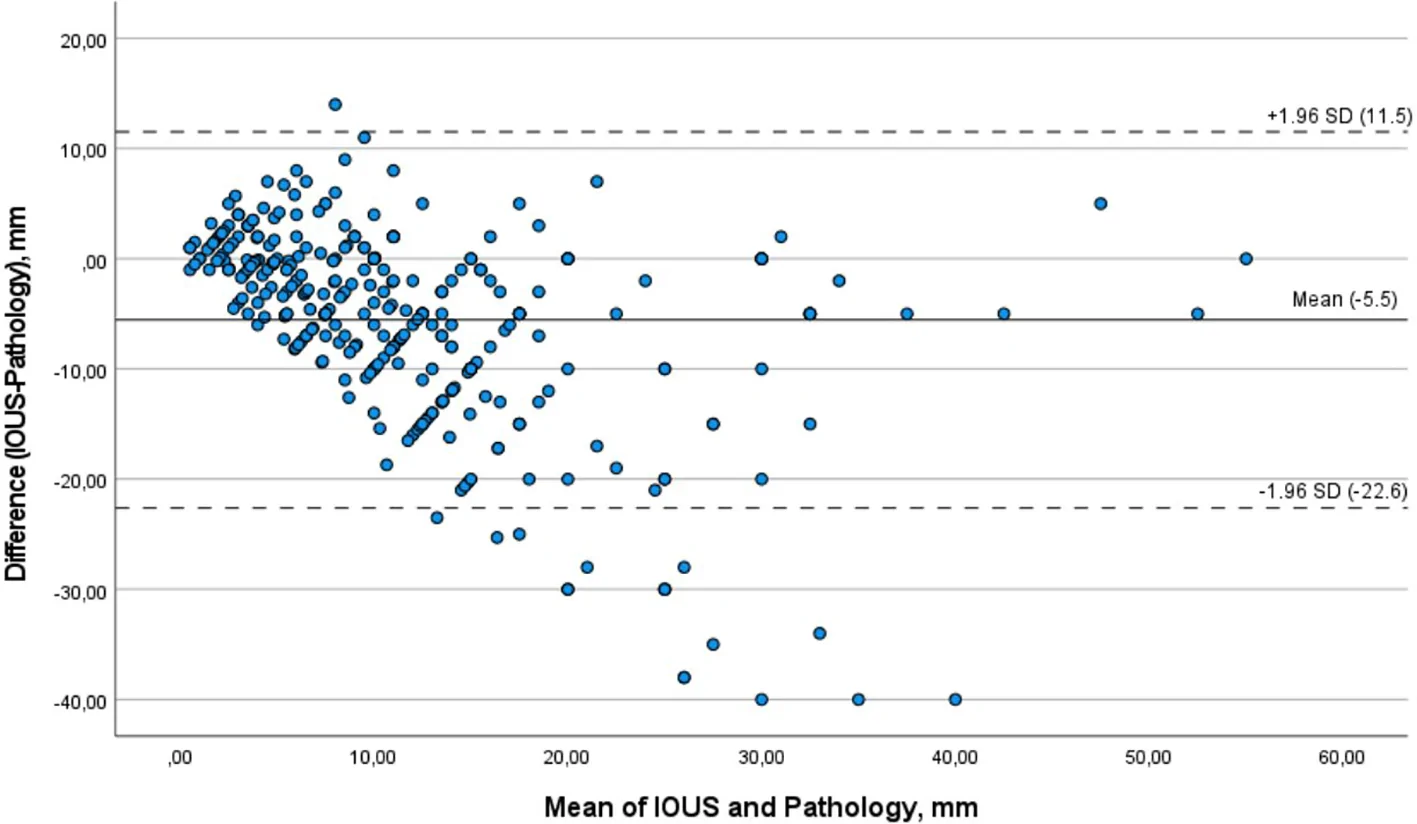
Bland-Altman analysis of agreement between intraoperative specimen ultrasonography and pathological margin measurements. Bland-Altman plot comparing intraoperative ultrasonography and paraffin margin measurements (360 margins). The solid central line indicates the mean difference (bias, -5.5 mm) and the dashed outer lines the 95% limits of agreement (-22.6 to +11.5 mm). The size-dependent spread (regression of difference on mean, p < 0.001) indicates closer agreement at narrow, clinically relevant margins. Fixed limits of agreement are shown for reference; the regression-based (size-dependent) limits are reported in the text.

IOUS underestimated the paraffin margin overall (mean difference −5.5 mm; 95% limits of agreement −22.6 to +11.5 mm), but this discrepancy was strongly size-dependent (regression of difference on mean, *p <0.001*): the estimated difference was less than 1 mm at margins near the 2 mm decision threshold and increased only for wide margins (approximately −9.6 mm at a 20 mm mean margin). The intraclass correlation coefficient (ICC) indicated moderate agreement for the point estimate (ICC = 0.508); however, its wide 95% confidence interval (0.239–0.672) spanned from poor to moderate agreement, and this uncertainty should be considered when interpreting the level of agreement (two-way mixed-effects model, absolute agreement, single measures; *p <0.001*). Agreement between IOUS and pathology was therefore closest in the clinically relevant range of narrow margins (**Figure 3**).

## Discussion

The adoption of BCS into routine clinical practice has established adequate surgical margin clearance and the use of RT as essential components for successful outcomes (12). Although the consensus defines “no ink on tumor” as an adequate margin, approaches may vary across countries and institutions (9). Bundred et al. suggested that achieving a minimum clear margin of 1 mm may be necessary to minimize both local and distant recurrence, thereby challenging the current “no ink on tumor” standard. This recommendation is supported by evidence showing that positive surgical margins following BCS are associated with significantly higher rates of both local and distant recurrence. Large-scale data indicate that patients with tumors on inked margins have a distant recurrence rate of approximately 25.4%, compared with 7.4% in those with negative margins, along with a nearly twofold increase in the risk of local recurrence (HR 1.98). Involved or close margins are also associated with worse outcomes, with a distant recurrence rate of 8.4% for the combined ‘tumor on ink or close’ category, and increased risks of both local (HR 2.09) and distant recurrence (HR 1.38) compared with wider margins (13).

With the incorporation of oncoplastic surgery into routine clinical practice, larger resection volumes can be achieved while preserving cosmetic outcomes, leading to improved local control and reduced re-excision rates. Nevertheless, conventional BCS remains necessary, particularly in early-stage BC or level 1 procedures, where tumors are small and can be adequately managed with limited excision (14). Surgical margins can currently be assessed on a case-by-case basis using intraoperative imaging or pathological evaluation, and these approaches are routinely applied in clinical practice (10). The use of intraoperative pathological margin assessment, regardless of the specific technique employed, has been shown to reduce re-excision rates to approximately 10% following BCS (15). However, because BCS is performed across all levels of healthcare institutions, access to dedicated intraoperative margin assessment techniques may be limited in certain settings, which motivated the present evaluation of IOUS as a widely available, surgeon-operated alternative.

Several methodological considerations regarding the choice of reference standard warrant consideration. Although a positive margin is conventionally defined by the “no ink on tumor” criterion, only five patients (8.3%) exhibited tumor at the inked surface in the present cohort. Basing the primary accuracy analysis on such a limited number of events would have produced unstable estimates and correspondingly wide confidence intervals, making a reliable comparison between IOUS and intraoperative pathological assessment difficult. Furthermore, IOUS is a continuous distance measurement and almost never reads exactly 0 mm; judging it against a strict 0 mm reference therefore unfairly lowers its apparent accuracy. Accordingly, a close margin (<2 mm) was pre-specified as the primary reference for diagnostic accuracy (a threshold concordant with the optimal cut-off derived from ROC analysis), whereas the positive-margin definition was retained as a secondary, clinically relevant endpoint. This reflects how IOUS is actually used in surgery: the goal is not to detect ink-on-tumor itself, but to identify margins that are close enough to require intraoperative re-excision. The low positive-margin rate is nonetheless a limitation, and this analysis should be seen as exploratory until confirmed in a larger cohort.

When ultrasonographic and pathological margin distances were compared, IOUS consistently measured smaller values than pathology. This difference was small for narrow margins and became larger only for wide margins, a pattern reflected in the moderate ICC and Bland-Altman analysis. This underestimation is likely attributable to tissue deformation, probe compression, fixation-related shrinkage, and differences in measurement orientation between the two methods, and may also be influenced by surgical technique and the volume of tissue excised. Accordingly, IOUS should not be regarded as a direct millimeter-by-millimeter substitute for pathological measurement; rather, its clinical value lies in threshold-based intraoperative decision-making. At the 2 mm threshold, IOUS was more specific but less sensitive than the pathologist’s assessment, and the difference between the two did not reach statistical significance. This absence of a significant difference should not be interpreted as evidence of equivalent performance: the study was designed and powered to estimate the diagnostic accuracy of IOUS against permanent pathology, not to formally test equivalence or non-inferiority, and the number of discordant close-margin events was small, so a clinically meaningful difference cannot be excluded. While intraoperative pathological assessment remained superior for detecting close margins in our cohort, IOUS produced fewer false positives and may therefore serve as a useful alternative in settings where immediate pathological assessment is not available, reducing the number of second procedures compared with no intraoperative assessment at all.

A review of the literature shows that most studies on IOUS focus on US-guided BCS and its impact on re-excision rates. In these studies, IOUS has been associated with improved margin clearance and reduced re-excision rates, with reported re-excision rates as low as 6% compared to 17% with palpation-guided surgery. Furthermore, high rates of negative margins at initial surgery (91–93%) and low rates of final positive margins (approximately 2–4%) have been reported, supporting the effectiveness of US-guided techniques in achieving adequate resections (16). In a study of 45 patients, a 5-mm threshold was used for IOUS margin assessment, yielding sensitivity and specificity of 25% and 95%, respectively. While this low sensitivity indicates limited ability to detect inadequate margins, the high specificity suggests that margins ≥5 mm on US reliably predict negative pathological margins. Consistent with these findings, our study also demonstrated a similar pattern, with high NPV and limited positive predictive performance, supporting the role of specimen US primarily as a rule-out tool rather than a definitive indicator of margin positivity (17). In an analysis of 155 patients with both ductal carcinoma *in situ* and invasive ductal carcinoma, IOUS was used to assess the closest tumor–margin distance and was compared with pathological measurements. The findings suggest that margins measuring less than 0.5 cm on IOUS may require additional resection in the corresponding direction. Although IOUS can aid intraoperative margin assessment, it may overestimate the true margin distance and may fail to detect small multifocal lesions (18). Alam et al. reported that surgeon-performed specimen US provides workflow-efficient margin assessment with diagnostic performance comparable to radiologist-performed US, supporting its feasibility in resource-limited settings consistent with our findings. However, while they reported a strong correlation between ultrasonographic and histopathological margins (r = 0.87), our analysis demonstrated greater measurement variability, likely reflecting differences in tissue compression, specimen handling, and the number of margins assessed per specimen. Importantly, unlike prior specimen-level analyses based on very few margin-positive events, our study incorporated both patient-level and margin-level approaches, adjusted for within-patient correlation, providing a more robust estimate of diagnostic performance (19).

Several limitations of this study should be considered when interpreting the findings. A principal limitation was the compressibility effect during measurement, which may lead to underestimation of margin distances and can be influenced by tumor size, tissue elasticity, and the volume of the excised specimen. The cohort was dominated by luminal-type tumors, with 56 (93.3%) hormone receptor–positive/HER2-negative and only 3 HER2-amplified and 1 triple-negative tumor. This reflects the composition of an upfront breast-conserving surgery cohort, in which hormone receptor–positive tumors predominate, whereas HER2-positive and triple-negative tumors are more frequently managed with neoadjuvant systemic therapy and thus less often undergo upfront conservative surgery. For this reason, and because of the limited number of positive-margin events, subgroup analyses by molecular subtype, histologic subtype or tumor size could not be performed. The relatively small number of close margins (n = 25) limits the precision of these estimates; accordingly, the comparison with intraoperative pathological assessment was exploratory, with no pre-specified equivalence margin and no sizing to exclude a clinically meaningful difference. In addition, the optimal cutoff value may vary depending on the number of margins evaluated and whether measurements are performed by a single surgeon, and diagnostic performance may improve with further standardization.

In conclusion, IOUS showed a statistically significant association with the paraffin margin at both the patient and margin levels, supporting its value as a practical adjunct for margin assessment during BCS. Although its sensitivity at the 2 mm threshold was limited, IOUS functioned reliably as a rule-out tool at higher distance thresholds, where the negative predictive value was highest, and may be particularly useful where immediate intraoperative pathological assessment is not readily available. In such resource-limited or time-constrained settings, surgeon-performed US offers a rapid, accessible means of identifying margins that are likely to be clear, allowing more informed intraoperative decision-making. While its moderate positive predictive value indicates that it cannot fully replace definitive histopathological evaluation, IOUS represents a valuable complementary technique that may support the surgical workflow where conventional methods are limited.

## Data Availability

The raw data supporting the conclusions of this article will be made available by the authors, without undue reservation.
